# JMJD2C-mediated long non-coding RNA MALAT1/microRNA-503-5p/SEPT2 axis worsens non-small cell lung cancer

**DOI:** 10.1038/s41419-022-04513-5

**Published:** 2022-01-19

**Authors:** Jun Zhang, Mingliang Wang, Jiashun Wang, Wendong Wang

**Affiliations:** Department of Thoracic Surgery, Union Hospital, Tongji Medical College, Huazhong University of Science and Technology, 430022 Wuhan, China

**Keywords:** Cancer, Diseases

## Abstract

Jumonji domain containing protein 2C (JMJD2C) could epigenetically regulate cancer cells. We specifically explored the downstream mechanism of JMJD2C in non-small cell lung cancer (NSCLC) from the long non-coding RNA metastasis associated with lung adenocarcinoma transcript 1/microRNA-503-5p/septin 2 (MALAT1/miR-503-5p/SEPT2) axis. NSCLC clinical tissues were utilized to assess JMJD2C, MALAT1, miR-503-5p and SEPT2 levels. NSCLC cell lines (A549 and H1299) were applied for loss-of-function and gain-of-function tests to identify the functional roles of JMJD2C, MALAT1, miR-503-5p, and SEPT2. The interactions among JMJD2C, MALAT1, miR-503-5p, and SEPT2 were assessed. Augmented JMJD2C, MALAT1, and SEPT2 and reduced miR-503-5p levels were found in NSCLC. Depleting JMJD2C or MALAT1, or restoring miR-503-5p exerted anti-tumor effects on NSCLC cells in vitro and in vivo. JMJD2C is bound to the promoter of MALAT1. MALAT1 bound to miR-503-5p and miR-503-5p targeted SEPT2. Knocking down MALAT1 or SEPT2, or elevating miR-503-5p mitigated the pro-tumor effects of upregulated JMJD2C on NSCLC. It is evident that the JMJD2C-mediated MALAT1/miR-503-5p/SEPT2 axis takes part in the process of NSCLC and even worsens NSCLC.

## Introduction

Lung cancer is composed of subpopulations of cells or clones with different molecular characteristics, resulting in intratumoral heterogeneity [1]. Non-small cell lung cancer (NSCLC) takes the majority of lung cancer cases, of which about a quarter are classified as locally advanced disease, otherwise known as stage III disease [2]. Late diagnosis of lung cancer primarily blames on asymptom in early-stage, and misdiagnosis with early symptoms [3]. Stage-based treatments vary in patients with NSCLC which are comprised of complete surgical resection, conventional, or stereotactic radiotherapy, and percutaneous thermal ablation [4]. Having a thorough insight into the mechanism of NSCLC could facilitate to managing the disease.

JMJD2 histone demethylases are thought to be epigenetic mediators in cancers [5] and JMJD2 overexpression has oncogenic potential [6]. In addition, JMJD2s have been reported to dramatically correlate with cisplatin resistance in NSCLC [7]. Concerning JMJD2C, some research has pointed out that it drives cancer migration and invasion in lung cancer [8] and mediates tumorigenesis in osteosarcoma [9]. Given that JMJD2C promotes colorectal cancer (CRC) metastasis via histone methylation of metastasis-related lung adenocarcinoma transcript 1 (MALAT1) [10], it is unclear whether and how JMJD2C-mediated downstream involving MALAT1 functions in NSCLC. MALAT1 has been identified as an onco-lncRNA involved in the promotion of NSCLC [11]. It is noted that MALAT1 confers acquired malignant phenotypes of lung cancer cells [12, 13], as well as enhanced drug resistance for NSCLC cells by cooperation with microRNAs [14]. Altered miRNA expression profiles have diagnostic value and can even program individualized therapy for NSCLC [15]. As a member of miRNA, miR-503 has the capacity to retard NSCLC [16] and mediate cisplatin resistance [17]. miR-503-5p is an inhibitory effector in ovarian cancer that could be sponged by MALAT1 [18]. Based on that, we would like to know whether MALAT1/miR-503-5p works in NSCLC cell progression. Septins (SEPTs) are connected to the cytosolic actin cytoskeleton in matrix-attached cancer cells and act critically in metastatic cancer cells [19]. Through bioinformatics software, we found that SEPT2 was a predicted target of miR-503-5p and studies have partially explored SEPT2-related mechanisms in cancers from its feedback with lncRNA-miRNA [20, 21].

Thus, we conducted the research with the hypothesis that JMJD2C-mediated histone methylation of MALAT1 stimulates the progression of NSCLC by interacting with miR-503-5p and SEPT2.

## Methods and materials

### Ethics statement

All patients signed an informed consent form. Experimental approval was obtained from the ethics committee of Union Hospital, Tongji Medical College, Huazhong University of Science and Technology. Animal research has been approved by the animal care and use Committee of Union Hospital, Tongji Medical College, Huazhong University of Science and Technology.

### Subjects

Tissue specimens (cancer tissues and adjacent tissues) were acquired from 116 patients with NSCLC in Union Hospital, Tongji Medical College, Huazhong University of Science and Technology. The patients including 69 males and 47 females were aged at 46–72 years old. All patients were diagnosed as NSCLC by histopathology and received no pre-operation treatments.

### Cell culture

Normal lung bronchial epithelial cell line (BEAS-2B) and lung cancer cell line (A549) were cultured in Dulbecco’s modified Eagle medium (DMEM; Gibco, CA, USA) while another lung cancer cell line (H1299) in Roswell Park Memorial Institute-1640 medium (Gibco). The above medium was supplemented with 10% fetal bovine serum (FBS; Gibco), penicillin (500 units/mL) and streptomycin (200 µg/mL). The cell lines were from BeNa culture collection (Beijing, China).

### Cell transfection

Transfection was conducted with Lipofectamine 3000 (Invitrogen, CA, USA) according to the manufacturer’s instructions. A549 and H1299 cells were transfected with sh-NC, sh-JMJD2C, sh-CTR(control shRNA with scrambled sequence), sh-MALAT1, mimic NC, miR-503-5p mimic, JMJD2C overexpression plasmids, JMJD2C overexpression plasmids + sh-MALAT1, JMJD2C overexpression plasmids + miR-503-5p mimic, or JMJD2C overexpression plasmids + sh-SEPT2. Shortly, cells were cultured in a 6-well plate at 4 × 10^5^ cells/mL and transfected at 80% cell confluence. The medium was replaced 6 h after transfection, and the cells were harvested 48 h later. Genepharma (Shanghai, China) was responsible for synthesis of various constructs.

### 3-(4, 5-dimethylthiazol-2-yl)-2, 5-diphenyltetrazolium bromide (MTT) assay

Cells after transfection were seeded into 96-well plates at 3000 cells/plate and cultured for 24, 48, and 72 h, respectively. Then, each well was added with 20 µL MTT (5 mg/mL) for 4 h, and the purple formazan precipitate was dissolved in 200 µL dimethyl sulfoxide (Sigma-Aldrich). Optical density_490nm_ was measured by a spectrophotometer and cell growth curve was drawn [22].

### Colony formation assay

Cells were counted after transfection, seeded into 6-well plates at 3000 cells/plate and cultured for 9–14 days until visible colonies appeared, during which the medium was refreshed every 48 h. Then, cells fixed in 75% ethanol were subjected to staining by 0.1% crystal violet. At least 50 cells were contained in each colony and viewed under ten fields of view [23].

### Flow cytometry

After 72 h of transfecion, cells were rinsed with PBS twice, immersed in binding buffer and combined with annexin V-fluorescein isothiocyanate (BD Biosciences, NJ, USA) and propidium iodide (BD Biosciences) in the darkness. Cell apoptosis was measured on a flow cytometer at 488 nm (Agilent, Hangzhou, China) [24].

### Transwell assay

Migration test: Cells after transfection were seeded in the upper chamber of the transwell containing 24-well plate in serum-free medium at 1 × 10^5^ cells/well. The lower chamber was filled with complete medium. Cells were incubated for 48 h, and those migrated under the membrane were fixed with 4% paraformaldehyde, and stained with 1% crystal violet for 20 min. The cells were sealed with neutral resin and observed in five random fields of view.

Invasion test: Matrigel was added to the membrane of the upper chamber, the rest steps were similar to the migration test [25].

### Reverse transcription quantitative polymerase chain reaction (RT-qPCR)

After being extracted from tissues and cells using Trizol kit (Invitrogen; Thermo Fisher Scientific, MA, USA), and quantified using NanoDrop ND-1000 (NanoDrop), total RNA was transformed into cDNA through Mir-X^TM^ miRNA First Strand Synthesis Kit (for miRNA) and Primpscript®RT reagent kit (for mRNA and lncRNA) (both Takara, Japan). PCR was amplified on the ABI PRISM 7700 (Applied Biosystems; Thermo Fisher Scientific) using SYBR Premix Ex Taq™ II PCR kit (Takara). Supplementary Table 1 showed primer sequences (U6 and glyceraldehyde-3-phosphate dehydrogenase [GAPDH] as internal controls). The 2^−ΔΔCt^ method was applied to quantitative analysis.

### Western blot assay

After lysis by radio-immunoprecipitation assay lysis solution (Beyotime), the tissues and cells were centrifuged and the supernatant was collected. The protein concentration was measured by using the BCA kit (Beyotime) The proteins (30 μg) were added with β-mercaptoethano, boiled for 10 min, and separated by sodium dodecyl sulphate polyacrylamide gel electrophoresis. Then, the protein transferred to a nitrocellulose membrane (Bio-Rad, CA, USA) was combined with the primary antibodies, anti-SEPT2 (1:500, Proteintech, USA), anti-JMJD2C (1:500), and anti-GAPDH (1:1000, both from Abcam, MA, USA), as well as the secondary antibody for reaction. The membrane was treated with enhanced chemiluminescence reagent (SuperSignal Western Pico Chemiluminescent Substrate; Pierce, USA)). Image J software (National Institutes of Health) was applied to protein quantitative analysis. The gray value of the band was normalized to GAPDH.

### Dual luciferase reporter gene assay

To evaluate the function of JMJD2C on MALAT1 promoter activity, A549 cells were transfected with sh-NC or sh-JMJD2C with a luciferase reporter plasmid containing MALAT1 promoter through Lipofectamine 3000 (Invitrogen). After 48 h, cell lysates were subjected to analysis of relative luciferase activity on the dual luciferase reporter gene detection system (Promega, WI, USA) [10].

The interaction between MALAT1 and miR-503-5p and the targeting relationship between miR-503-5p and SEPT2 were conformed. MALAT1 wild-type (WT), MALAT1 mutant (MUT), SEPT2 WT and SEPT2 MUT potentially binding to miR-503-5p were inserted into pGL4 luciferase reporter and co-transfected with miR-503-5p mimic or mimic-NC into A549 cells. After 48 h, the relative luciferase activity was measured using a dual luciferase detection system (Promega).

### Chromatin immunoprecipitation (ChIP) assay

ChIP assay kit (Millipore, MA, USA) was utilized. The chromatin extracted from cells were immunoprecipitated with anti-JMJD2C antibody (Abcam). A control immunoglobulin G (Merck, Germany) was applied. Primers of MALAT1 promoter: forward: 5′-GGTCAGCCTGAGACCACTTC-3′, reverse: 5′-CTGTGCCTGTTCTGGGGAAT-3′ [10, 26].

### RNA immunoprecipitation (RIP) assay

With Magna RIP™ RIP kit (Millipore), RIP analysis was carried out. Cells that were lysed in complete radio-immunoprecipitation assay (protease inhibitor cocktail and RNase inhibitor) were combined with RIP buffer containing magnetic beads coupled with human anti-Ago2 antibody (Millipore) or immunoglobulin G(IgG). The sample was added with proteinase K and RNase inhibitors, and isolation of immunoprecipitated RNA was performed. The purified RNA was analyzed by RT-qPCR [23].

### Tumor formation assay

BALB/c nude mice (5 weeks old) were employed for animal studies. A549 cells with stably JMJD2C-inhibition, MALAT1-inhibition, miR-503-5p-overexpression were established and injected subcutaneously into the left side of the posterior flank of nude mouse. Tumor growth was monitored by measuring tumor maximum length and minimum length every 7 days, and tumor volume was calculated by the formula: volume (mm^3^) = (width^2^ × length/2). On the 28th day, tumors were excised from euthanized mice [27].

### Statistical analysis

Data were analyzed with GraphPad Prism 6 (La Jolla, CA, USA). Fisher’s exact test, Kaplan–Meier survival analysis, Pearson correlation test *t*-test, and analysis of variance (ANOVA) were used to analyze data. *P* < 0.05 was considered statistically significant (*P < 0.05, **P < 0.01, ***P < 0.001).

## Results

### JMJD2C-related MALAT1 is highly expressed in NSCLC tissues; JMJD2C is related to NSCLC clinicopathological factors

**Fig. 1:**
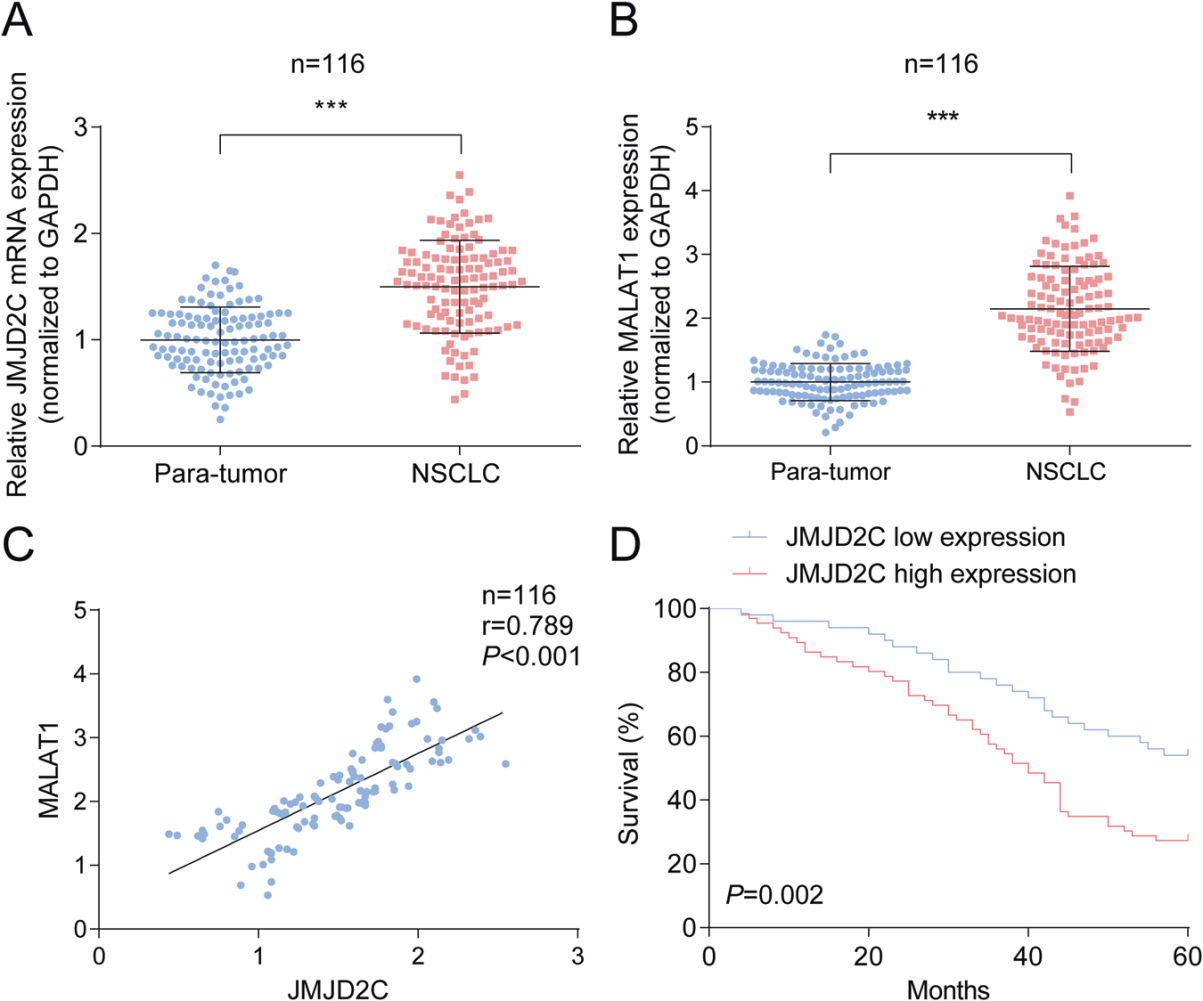
JMJD2C-related MALAT1 is highly expressed in NSCLC tissues. **A** RT-qPCR analysis of JMJD2C in NSCLC tissue; **B** RT-qPCR analysis of MALAT1 in NSCLC tissue; **C** Correlation between JMJD2C and MALAT1; **D** Relation between JMJD2C expression and survival of patients with NSCLC. ***P < 0.001; Data statistics was by *t*-test, Pearson’s correlation analysis, Kaplan–Meier analysis.

The differences in levels of JMJD2C, MALAT1 in NSCLC tumor tissues and para-tumor tissues were assessed by RT-qPCR. As indicated by the outcome, JMJD2C and MALAT1 were highly expressed in NSCLC tissues compared with para-tumor tissues (Fig. 1A, B). Subsequently analyzed by Pearson test, we found the positive correlations between levels of JMJD2C and MALAT1 in tumor tissues (Fig. 1C).

For further analysis of the clinical significance of JMJD2C in NSCLC, we divided NSCLC patients into JMJD2C high expression and JMJD2C low expression groups (Table 1) and found that JMJD2C expression was associated with tumor size and tumor node metastasis. Also, after Kaplan–Meier analysis, we determined that patients with high JMJD2C expression had shorter overall survival (Fig. 1D).

**Table 1:** The relationship between JMJD2C expression and clinicopathological characteristics in patients with NSCLC.

Parameter	Case	JMJD2C		*P* value
		Low expression (*n* = 50)	High expression (*n* = 66)	
Age (years)				0.254
<60	44	22	22	
≥60	72	28	44	
Gender				0.447
Male	69	32	37	
Female	47	18	29	
Smoking history				0.457
Yes	56	22	34	
No	60	28	32	
Tumor size				0.023
<3 cm	64	34	30	
≥3 cm	52	16	36	
Histology type				0.057
Adenocarinoma	70	25	45	
Squamous	46	25	21	
Lymph nodes metastasis				0.186
No	65	32	33	
Yes	51	18	33	
TNM stage				0.014
I–II	68	36	32	
III–IV	48	14	34	

### JMJD2C binds to the promoter of MALAT1

JMJD2C has great potential in the epigenetic regulation of tumors [28], especially in regulation of target gene promoter activity. We measured JMJD2C, MALAT1 level in cells through RT-qPCR and found higher JMJD2C, MALAT1 level in NSCLC cell lines (A549 and H1299) when compared with human lung bronchial epithelial cell line (BEAS-2B) (Supplementary Fig. 1A, B).

Next, we performed ChIP and discovered that in A549 cells, JMJD2C could bind to the promoter of MALAT1 (Supplementary Fig. 1C). Also, dual luciferase reporter gene test further confirmed that knocking down JMJD2C reduced the activity of MALAT1 promoter (Supplementary Fig. 1D). RT-qPCR test also showed that MALAT1 was downregulated after silencing JMJD2C (Supplementary Fig. 1E).

### Preventive effects of silenced JMJD2C on NSCLC cells

**Fig. 2:**
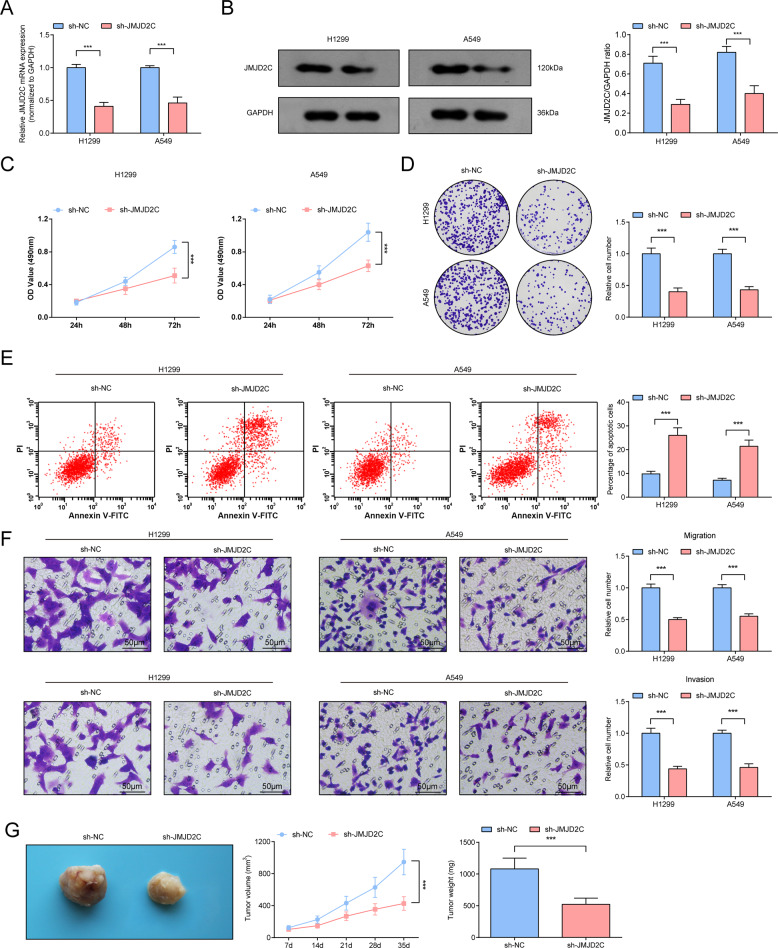
Preventive effects of silenced JMJD2C on NSCLC. **A**, **B** RT-qPCR and Western blot analysis of JMJD2C level after downregulating JMJD2C; **C** MTT measured the growth curve of A549 and H1299 cells after downregulating JMJD2C; **D** Colony formation assay measured colony formation ability of A549 and H1299 cells after downregulating JMJD2C; **E** Flow cytometry measured A549 and H1299 cell apoptosis after downregulating JMJD2C; **F** Transwell measured cell migration and invasion ability after downregulating JMJD2C; **G** Tumor formation assay evaluated tumor volume and weight after downregulating JMJD2C. ***P < 0.001; Data statistics was by *t*-test.

To thoroughly understand the biological function of JMJD2C, we downregulated JMJD2C expression in A549 and H1299 cells (Fig. 2A, B). Next, data collected from MTT, colony formation, Transwell and flow cytometry revealed that as to A549 and H1299 cells depleted of JMJD2C, viability, colony-forming, invasive and migratory properties were impaired and apoptosis was aggrandized (Fig. 2C–F).

Next, the carcinogenic potential of JMJD2C in vivo was uncovered through injecting A549 cells with stable and low expression of JMJD2C. The outcomes manifested that downregulating JMJD2C suppressed the growth of xenograft tumors (Fig. 2G).

Evidently, silencing JMJD2C suppressed cellular and tumor growth of NSCLC in vitro and in vivo.

### Inhibitory effects of downregulated MALAT1 on NSCLC cells

**Fig. 3:**
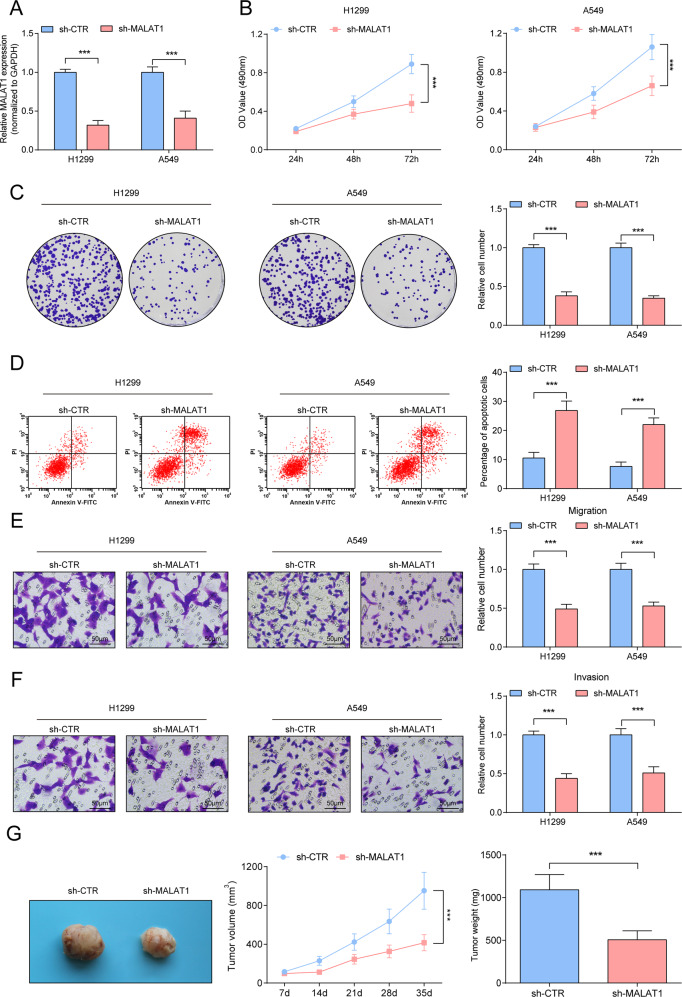
Inhibitory effects of down-regulated MALAT1 on NSCLC. **A** RT-qPCR analysis of MALAT1 level after down-regulating MALAT1; **B** MTT measured the growth curve of A549 and H1299 cells after downregulating MALAT1; **C** Colony formation assay measured colony formation ability of A549 and H1299 cells after downregulating MALAT1; **D** Flow cytometry measured A549 and H1299 cell apoptosis after downregulating MALAT1. **E**, **F** Transwell measured cell migration and invasion ability after downregulating MALAT1. **G** Tumor formation assay evaluated tumor volume and weight after downregulating MALAT1. ***P < 0.001; Data statistics was by *t*-test.

A549 and H1299 cells were successfully transfected with sh-MALAT1 to knock down MALAT1 expression (Fig. 3A). Subsequently, in A549 and H1299 cells with lowered MALAT1, the cellular malignant phenotypes were destructed (Fig. 3B–F). Moreover, in the xenograft nude mouse model, sh-MALAT1 reduced the growth of xenograft tumors (Fig. 3G).

### MALAT1 binds to miR-503-5p

miR-503-3p inhibits lung cancer cell viability and induces cell apoptosis [29]. Using RT-qPCR, we uncovered that miR-503-5p was downregulated in NSCLC cancer tissues and NSCLC cell lines (Supplementary Fig. 2A, B). Then we found that MALAT1 expression was negatively correlated with miR-503-5p level in tumor tissue (Supplementary Fig. 2C).

On the Starbase website, the binding sites between MALAT1 and miR-503-5p were found (Supplementary Fig. 2D). Then, in dual luciferase reporter gene detection, miR-503-5p mimic could reduce the luciferase activity of MALAT1-WT in A549 cells (Supplementary Fig. 2E). Also, RIP experiment tested the enriched MALAT1 and miR-503-5p levels in Ago2 immunoprecipitation (Supplementary Fig. 2F). Besides, RT-qPCR revealed an increment in miR-503-5p level in A549 and H1299 cells after downregulating MALAT1 (Supplementary Fig. 2G). Collectively, MALAT1 can specifically bind miR-503-5p, thereby regulating miR-503-5p level.

### Repressive effects of miR-503-5p on tumorigenicity of NSCLC

**Fig. 4:**
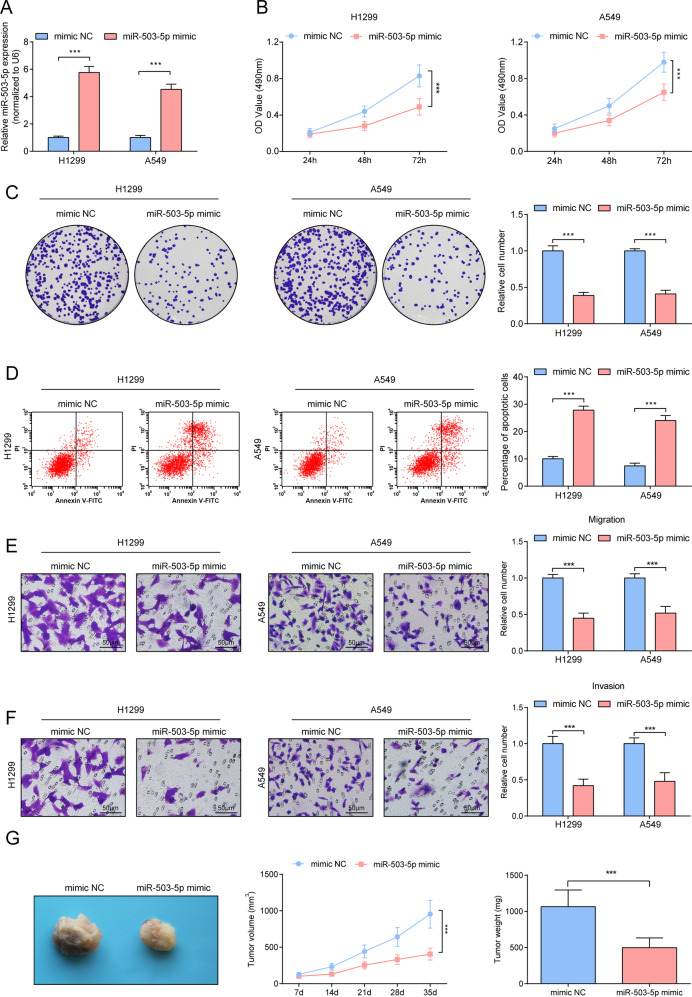
Repressive effects of miR-503-5p on tumorigenicity of NSCLC. **A** RT-qPCR analysis of miR-503-5p level after upregulating miR-503-5p. **B** MTT measured the growth curve of A549 and H1299 cells after upregulating miR-503-5p. **C** Colony formation assay measured colony formation ability of A549 and H1299 cells after upregulating miR-503-5p. **D** Flow cytometry measured A549 and H1299 cell apoptosis after upregulating miR-503-5p. **E**, **F** Transwell measured cell migration and invasion ability after upregulating miR-503-5p. **G** Tumor formation assay evaluated tumor volume and weight after upregulating miR-503-5p. ***P < 0.001; Data statistics was by *t*-test.

We transfected miR-503-5p mimic into A549 and H1299 cells, and verified the success of transfection by RT-qPCR (Fig. 4A). Notably, suppressed cellular progression was seen in A549 and H1299 cells up-regulating miR-503-5p (Fig. 4B–F). Also, in the xenograft nude mouse model, overexpression of miR-503-5p suppressed the growth of xenograft tumors (Fig. 4G).

### miR-503-5p negatively mediates SEPT2

SEPT2 level measured by RT-qPCR and Western blot was upregulated in NSCLC tissues and A549 and H1299 cells (Supplementary Fig. 3A, B). We found that SEPT2 expression was negatively correlated with miR-503-5p level in tumor tissue (Supplementary Fig. 3C). The targeting relationship between miR-503-5p and SEPT2 was predicted through the Starbase website (Supplementary Fig. 3D). The dual luciferase reporter gene test further examined that the luciferase activity of cells transfected with miR-503-5p mimic and SEPT2-WT was reduced (Supplementary Fig. 3E).

The outcome of Western blot also clarified that upregulating miR-503-5p in A549 and H1299 cells depressed SEPT2 level (Supplementary Fig. 3F), proving miR-503-5p targeting SEPT2.

### The pro-tumor effects of elevated JMJD2C on NSCLC cells are reversed by silencing MALAT1 or SEPT2, or restoring miR-503-5p

**Fig. 5:**
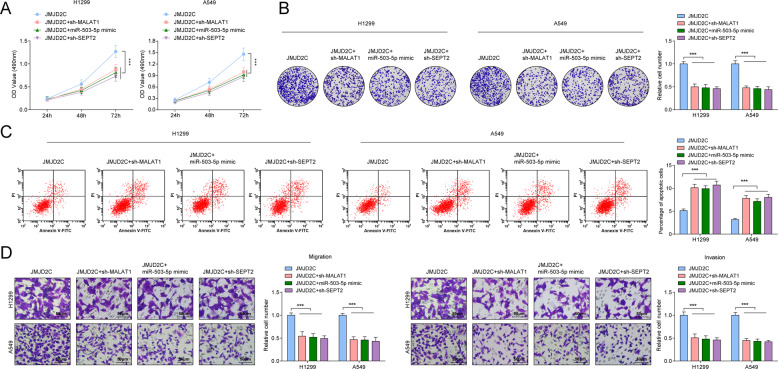
The promoting effects of elevated JMJD2C on NSCLC are reversed by silencing MALAT1 or SEPT2, or restoring miR-503-5p. **A** MTT measured the growth curve of A549 and H1299 cells. **B** Colony formation assay measured colony formation ability of A549 and H1299 cells. **C** Flow cytometry measured A549 and H1299 cell apoptosis. **D** Transwell measured cell migration and invasion ability; Data statistics was by *t*-test, ANOVA, Pearson’s correlation analysis.

The mechanism of JMJD2C, MALAT1, miR-503-5p, and SEPT2 in NSCLC was further assessed. We recommended sh-MALAT1, miR-503-5p mimic, or sh-SEPT2 into A549 and H1299 cells that had been transfected with JMJD2C overexpression plasmid. The pro-tumor effects of elevated JMJD2C on NSCLC cells were reversed by silencing MALAT1 or SEPT2, or restoring miR-503-5p (Fig. 5A–D).

In summary, JMJD2C mediated NSCLC by regulating MALAT1/miR-503-5p/SEPT2 axis.

## Discussion

**Fig. 6:**
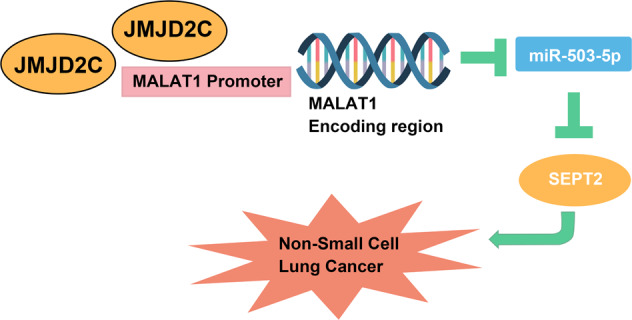
The network of JMJD2C. A schematic model of JMJD2C-mediated MALAT1/miR-503-5p/SEPT2 regulatory network in NSCLC.

NSCLC is caused by multi-step carcinogenesis induced by chemical and physical mutagens [30]. The molecular mechanism of NSCLC was partly explored from JMJD2C-regulated MALAT1/miR-503-5p/SEPT2 axis in our research. A summary was obtained that JMJD2C regulated the histone methylation of MALAT1, thus to upregulate MALAT1 level and deteriorate the malignant phenotypic of NSCLC cells via suppressing miR-503-5p and promoting SEPT2 (Fig. 6).

From the experimental data, we found that JMJD2C was upregulated in NSCLC that was connected with shorter survival time, and silencing JMJD2C reduced tumorigenicity of NSCLC. As a matter of fact, JMJD2C has been identified as the oncogene in pancreatic cancer, and deficiency of JMJD2C is capable to weaken cell proliferation and invasion [31]. The aberrant increment is seen in JMJD2C expression in osteosarcoma tissues that is suggested to associate with cell metastasis, and JMJD2C could drive the growth of osteosarcoma cells through inducing fibroblast growth factor 2 [9]. In the context of lung cancer, the elevated level of JMJD2C indicates an association with metastasis, and suppression of JMJD2C partly attributes to the impairments in migration and invasion, as well as tumor hepatic metastasis and epithelial–mesenchymal transition [8]. Apart from that, the augmented level of JMJD2C has been implied to supply the niche for colon cancer cell growth, but knockout of JMJD2C oppresses cell growth and colony forming ability [28], as well as retards lung metastasis in breast cancer [32]. Showing the consistence with these reports, JMJD2C adversely performed in NSCLC.

MALAT1 has been ensured to be positively regulated by JMJD2C through modification of histone methylation, and works to enhance metastasis of colorectal cancer cells [10]. Consistently, we discovered the mechanism that JMJD2C upregulated MALAT1 through mediating the methylation modification of H3K9 and H3K36 on the promoter of MALAT1. Previously proved, MALAT1 acts crucially in regulating the metastatic phenotype of lung cancer cells, and lung cancer cells deficient of MALAT1 are impaired in terms of their migration and metastasis abilities in xenografted tumors [12]. In NSCLC, the raised level of MALAT1 is detectable in NSCLC [33], and knocking out MALAT1 weakens the biological functions and tumorigenic ability of NSCLC cells through binding to miR-185-5p [25]. MALAT1 expression shows a reduction in NSCLC clinical samples, and elevated level of MALAT1 fuels the proliferation, migration and invasion activities of NSCLC cells [34].

Our discovery revealed that MALAT1 drove the development in NSCLC through interacting with the specific miRNA, miR-503-5p. Supported by a late report, MALAT1 targets miR-503-5p, thus to induce proliferation and suppress apoptosis of ovarian cancer cells [18]. Extensive reports have discussed that miR-503-5p represses tumors, complying our findings that elevating miR-503-5p hampered NSCLC cell growth in vitro and in vivo. In colon cancer, the success in suppressing tumorigenesis and lymphangiogenesis could be partly attributed to introduction of elevated miR-503-5p [35]. In addition, as to hepatocellular carcinoma cells introduced with upregulated miR-503-5p, their mobility and metastasis are impaired [36]. Mechanistically, miR-503-5p demonstrates a reduced level in osteosarcoma, and if suppressing miR-503-5p in osteosarcoma cells, the tumorigenesis progression could be push forward [37]. Overall, targeted restoration of miR-503-5p could restrain the malignant progression in tumors.

SEPT2 was a target of miR-503-5p and downregulating SEPT2 was proved to delay tumorigenesis in NSCLC. SEPT2 has been implicated to aggrandize the aggressiveness of various tumors, and silencing of SEPT2 is inhibitory for tumor growth. Evidently, downregulating SEPT2 restrains the invasive capability of glioblastoma cells [38] and the proliferative ability of epithelial ovarian cancer cells [39]. Similar to our findings, SEPT2 has been revealed to exert as a number of lncRNA-miRNA-mRNA network, thus to encourage growth of tumors. For instance, enhanced cellular proliferation in prostate cancer is triggered by LINC00473/miR-195-5p-regulated SEPT2 upregulation [40].

The present research for the first time discusses the mechanism of JMJD2C-mediated MALAT1/miR-503-5p/SEPT2 axis in NSCLC. To conclude briefly, the investigation explained that JMJD2C drives the progression of NSCLC through modifying histone methylation of MALAT1 to upregulate miR-503-5p-targeted SEPT2, supplying a possible chance to control NSCLC. Due to laboratory conditions, animal models of tumor metastasis were not used to detect the effect of the JMJD2C/MALAT1/miR-503-5p/SEPT2 signal axis on NSCLC in vivo. Also, the expression and survival analysis of JMJD2C, MALAT1, miR-503-5p, and SEPT2 was not provided to the public database due to resource constraints.

## Supplementary information

Supplementary Figure 1

Supplementary Figure 2

Supplementary Figure 3

Supplementary table 1

Supplementary Figure legend

author-contribution-form

aj-checklist

## Data Availability

Not applicable.
