# Supplementary figures and images for: JMJD2C-mediated long non-coding RNA MALAT1/microRNA-503-5p/SEPT2 axis worsens non-small cell lung cancer
Source: Cell Death Dis. 2022 Jan 19;13(1):65. doi: 10.1038/s41419-022-04513-5 (PMC8770565; doi:10.1038/s41419-022-04513-5)

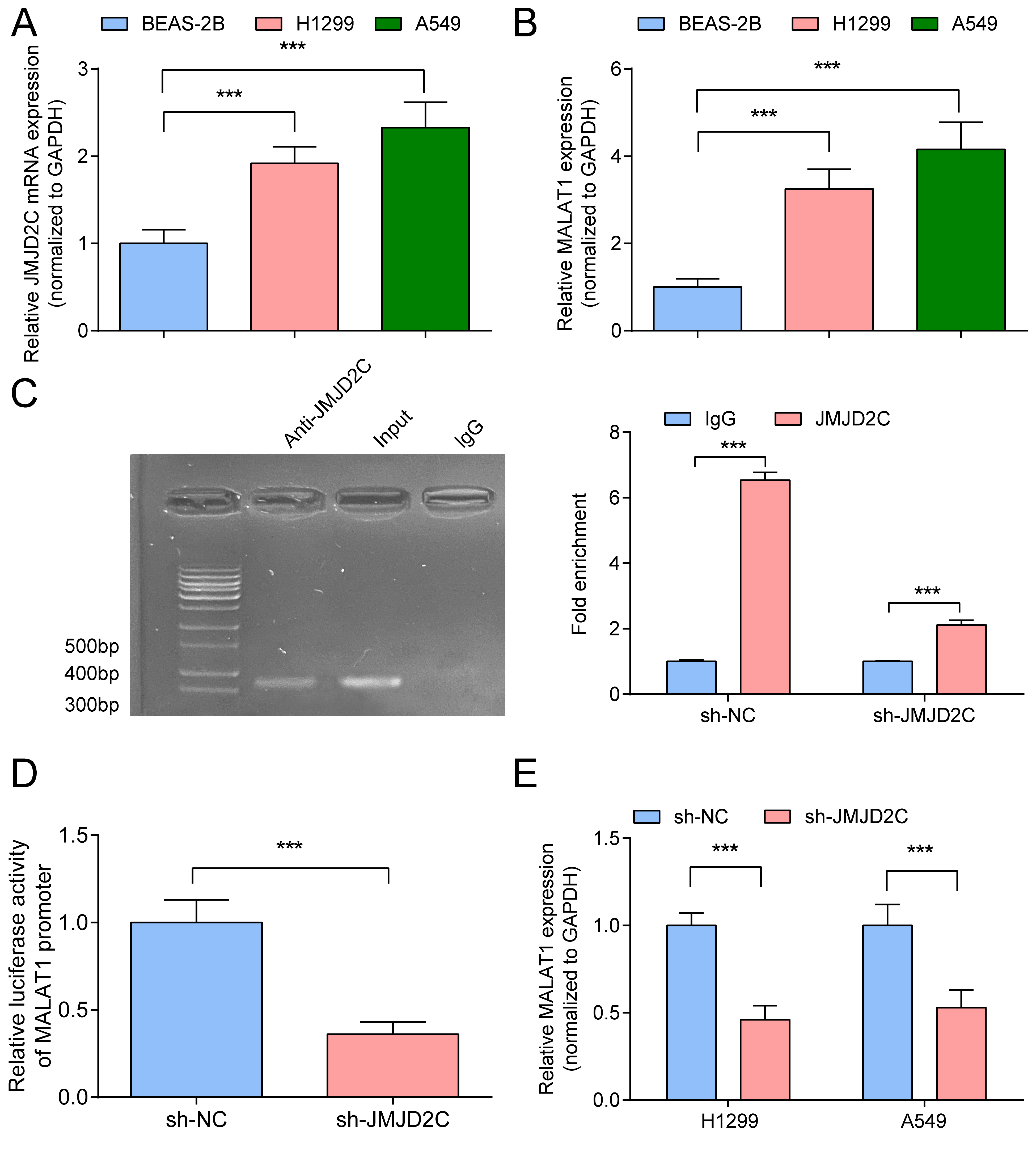

Supplement: Supplementary file 1 — Supplementary Figure 1 [file 41419_2022_4513_MOESM1_ESM.tif]

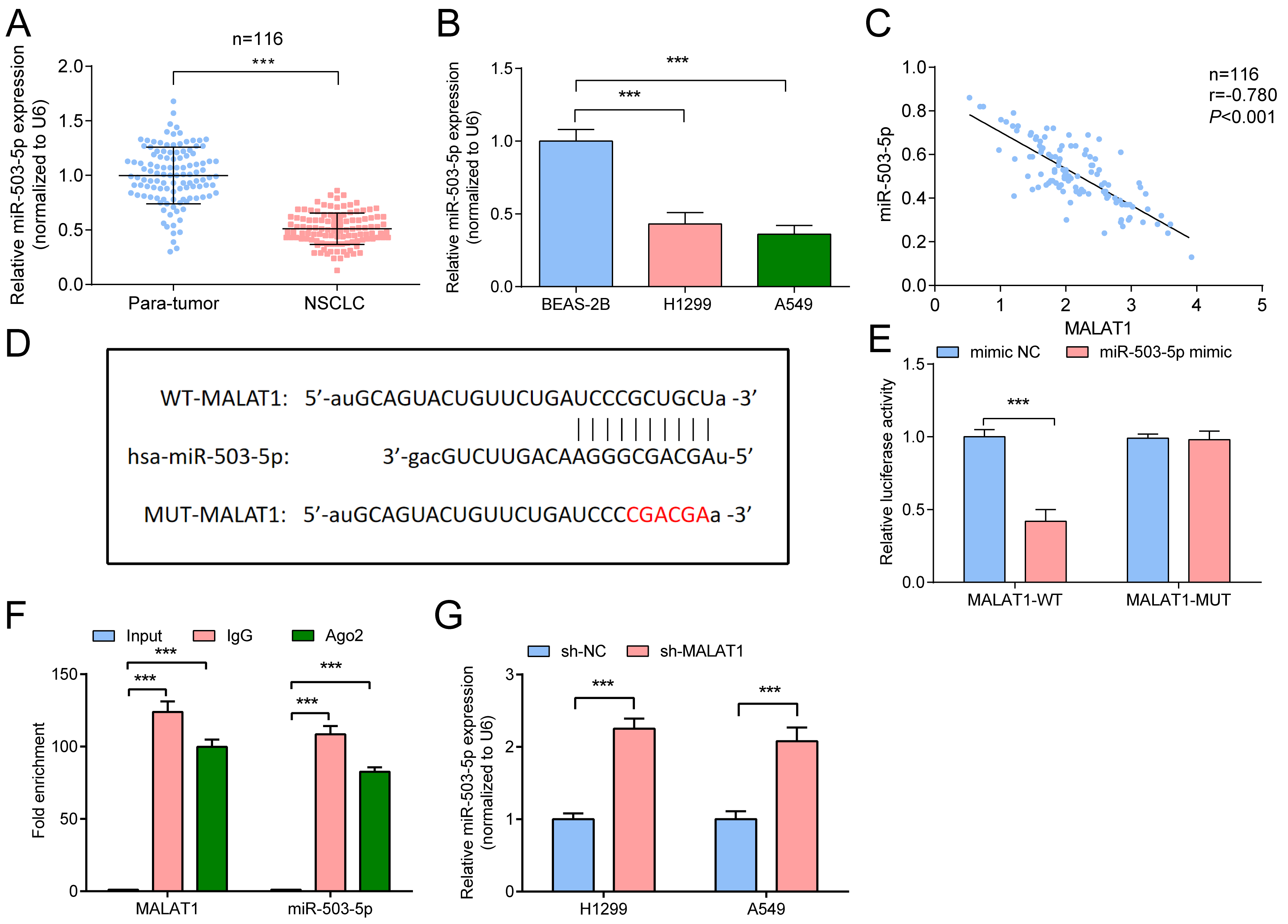

Supplement: Supplementary file 2 — Supplementary Figure 2 [file 41419_2022_4513_MOESM2_ESM.tif]

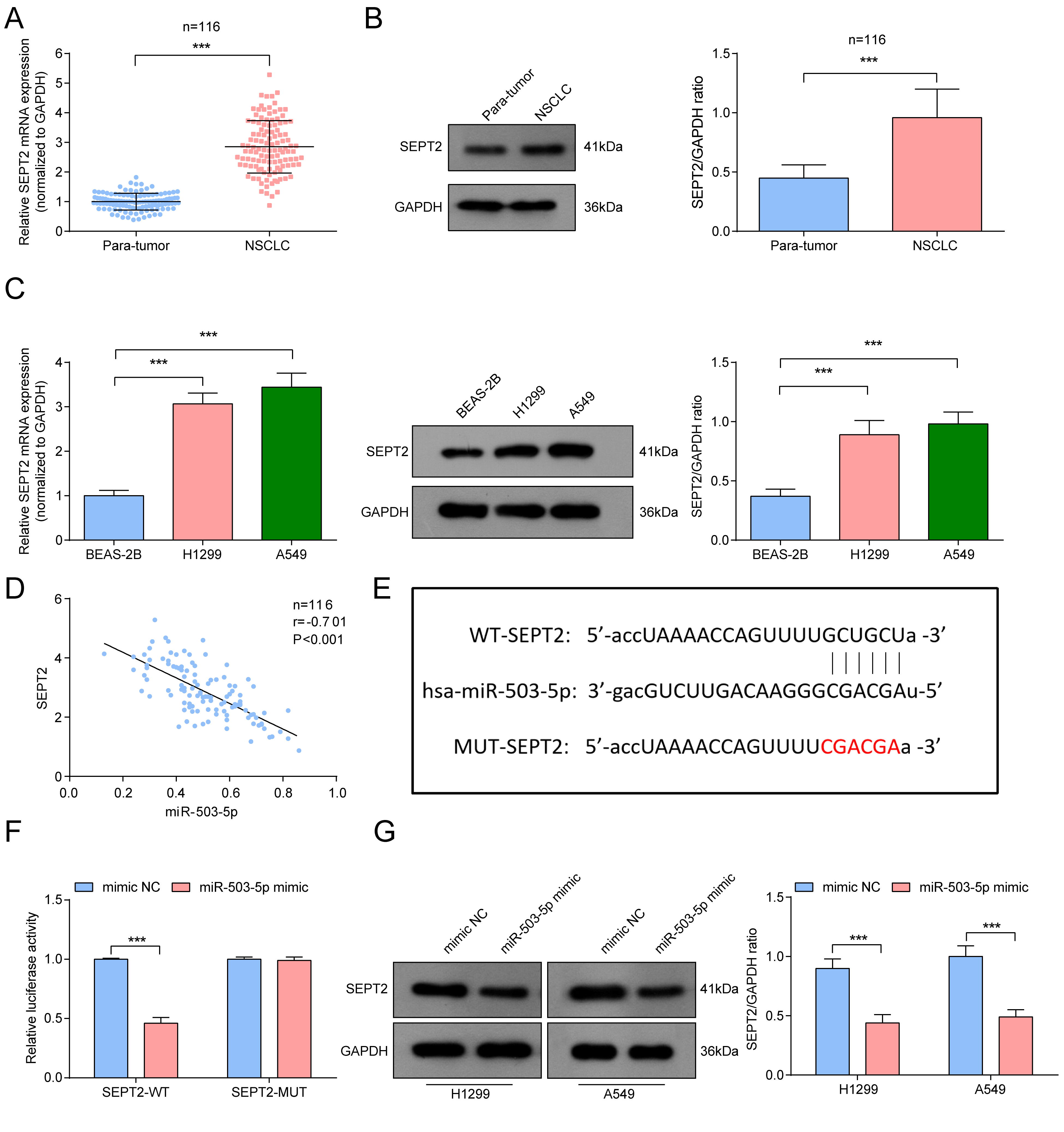

Supplement: Supplementary file 3 — Supplementary Figure 3 [file 41419_2022_4513_MOESM3_ESM.tif]
