# Supplementary material for: JMJD2C-mediated long non-coding RNA MALAT1/microRNA-503-5p/SEPT2 axis worsens non-small cell lung cancer
Source: Cell Death Dis. 2022 Jan 19;13(1):65. doi: 10.1038/s41419-022-04513-5 (PMC8770565; doi:10.1038/s41419-022-04513-5)
Supplement: Supplementary file 4 — Supplementary table 1 [file 41419_2022_4513_MOESM4_ESM.docx]

**Supplementary table 1** Primer sequences for RT-qPCR

| Gene | Primers (5’– 3’) |
| --- | --- |
| JMJD2C | Forward: GCTTGCGAGAAGGTCATTTC |
|  | Reverse: AGACAGTCTCGGCTCACGAT |
| MALAT1 | Forward: GAATTGCGTCATTTAAAGCCTAGTT |
|  | Reverse: GTTTCATCCTACCACTCCCAATTAAT |
| SEPT2 | Forward: GGTGACGCTATCAACTGCAGAG |
|  | Reverse: ATGATGTGCCGCCTGTTCAAGC |
| GAPDH | Forward: GAAGGTGAAGGTCGGAGTC |
|  | Reverse: GAAGATGGTGATGGGATTTC |
| MiR-503-5p | Forward: TAGCAGCGGGAACAGTTCTGCAG |
| U6 | Forward: CTCGCTTCGGCAGCACA |

Note: JMJD2C, Jumonji domain containing protein 2C; MALAT1, Long non-coding RNA metastasis associated lung adenocarcinoma transcript 1; MiR-503-5p, MicroRNA-503-5p; SEPT2, Septin 2; GAPDH, glyceraldehyde-3-phosphate dehydrogenase.
