# Supplementary material for: JMJD2C-mediated long non-coding RNA MALAT1/microRNA-503-5p/SEPT2 axis worsens non-small cell lung cancer
Source: Cell Death Dis. 2022 Jan 19;13(1):65. doi: 10.1038/s41419-022-04513-5 (PMC8770565; doi:10.1038/s41419-022-04513-5)
Supplement: Supplementary file 5 — Supplementary Figure legend [file 41419_2022_4513_MOESM5_ESM.docx]

**Supplementary Figure 1** JMJD2C binds to the promoter of MALAT1. A. RT-qPCR analysis of JMJD2C in NSCLC cell lines; B. RT-qPCR analysis of MALAT1 in NSCLC cell lines; C. ChIP detection of the interaction between JMJD2C and MALAT1 promoter; D. Luciferase detection of MALAT1 promoter activity; E. RT-qPCR analysis of MALAT1 expression after down-regulating JMJD2C. *** *P* < 0.001; Data statistics was by one-way ANOVA and *t-test*.

**Supplementary Figure 2** MALAT1 binds to miR-503-5p. A. RT-qPCR analysis of miR-503-5p level in NSCLC tissue; B. RT-qPCR analysis of miR-503-5p level in NSCLC cell line; C. Correlation between miR-503-5p and MALAT1; D. Starbase website predicted the target sites of miR-503-5p and MALAT1; E. Luciferase reporter gene experimental verified targeting relationship between MALAT1 and miR-503-5p; F. RIP detected the binding of MALAT1 and miR-503-5p; G. RT-qPCR analysis of miR-503-5p after down-regulating MALAT1. *** *P* < 0.001; Data statistics was by one-way ANOVA and Pearson's correlation analysis.

**Supplementary Figure 3** miR-503-5p negatively mediates SEPT2. A. RT-qPCR and Western blot analysis of SEPT2 in NSCLC tissue; B. RT-qPCR and Western blot analysis of SEPT2 in NSCLC cells line; C. Correlation between miR-503-5p and SEPT2;D. Starbase website predicted the target sites of miR-503-5p and SEPT2; E. Luciferase reporter gene experimental verified targeting relationship between miR-503-5p and SEPT2; F. Western blot analysis of SEPT2 after up-regulating miR-503-5p. *** *P* < 0.001; Data statistics was by t-test, ANOVA, Pearson's correlation analysis.
