# Supplementary material for: JMJD2C-mediated long non-coding RNA MALAT1/microRNA-503-5p/SEPT2 axis worsens non-small cell lung cancer
Source: Cell Death Dis. 2022 Jan 19;13(1):65. doi: 10.1038/s41419-022-04513-5 (PMC8770565; doi:10.1038/s41419-022-04513-5)
Supplement: Supplementary file 6 — author-contribution-form [file 41419_2022_4513_MOESM6_ESM.pdf]

DECLARATION OF CONTRIBUTIONS TO ARTICLE

ADMC

Manuscript Number:	Journal Name:	
CDDIS-21-1154R	*Cell Death Disease*	(the 'Journal')

Proposed Title of the Contribution:

study design；experimental studies； data analysis； manuscript editing	(the 'Contribution)

Author(s):

Jun Zhang, Mingliang Wang, Jiashun Wang, Wendong Wang	(the 'Authors')

For all *CDDis*covery articles, each person named as an author in the published version must be able to show he or she has contributed substantially to the article.

Authorship credit should be based on 1) substantial contributions to conception and design, acquisition of data, or analysis and interpretation of data; 2) drafting the article or revising it critically for important intellectual content; and 3) final approval of the version to be published. Authors should meet conditions 1, 2 and 3.

Any person who cannot be shown to have made a substantial contribution to the article cannot be listed as an author in the final version. The name of any person who is deemed to have made a minor contribution can, however, appear in the Acknowledgments section of the article.

Please complete the table below to indicate the contributions of all named authors to the manuscript.

Author Full Name:	Specification of Contribution to the Manuscript:
Jiashun Wang, Wendong Wang	finished study design
Mingliang Wang	finished experimental studies
Jun Zhang, Mingliang Wang, Jiashun Wang	finished data analysis
Wendong Wang	finished manuscript editing
All authors	read and approved the final manuscript

Please complete the table below to indicate the contributions of all named authors to the figures.

Figure 1:

Jun Zhang, Mingliang Wang, Jiashun Wang, Wendong Wang

Figure 2:

Jun Zhang, Mingliang Wang, Jiashun Wang, Wendong Wang

Figure 3:

Jun Zhang, Mingliang Wang, Jiashun Wang, Wendong Wang

Figure 4:

Jun Zhang, Mingliang Wang, Jiashun Wang, Wendong Wang

Figure 5:

Jun Zhang, Mingliang Wang, Jiashun Wang, Wendong Wang

Figure 6:

Jun Zhang, Mingliang Wang, Jiashun Wang, Wendong Wang
Supplementary Figure 1:Jun Zhang, Mingliang Wang, Jiashun Wang, Wendong Wang
Supplementary Figure 2:Jun Zhang, Mingliang Wang, Jiashun Wang, Wendong Wang
Supplementary Figure 3:Jun Zhang, Mingliang Wang, Jiashun Wang, Wendong Wang

Signed for and on behalf of the Author(s):	Print Name:	Date:
